# Increased aortic stiffness and elevated blood pressure in response to exercise in adult survivors of prematurity

**DOI:** 10.14814/phy2.14462

**Published:** 2020-06-19

**Authors:** Christopher R. Barnard, Matthew Peters, Amy L. Sindler, Emily T. Farrell, Kim R. Baker, Mari Palta, Harald M. Stauss, John M. Dagle, Jeffrey Segar, Gary L. Pierce, Marlowe W. Eldridge, Melissa L. Bates

**Affiliations:** ^1^ Department of Health and Human Physiology University of Iowa Iowa City IA USA; ^2^ Department of Pediatrics University of Wisconsin Madison WI USA; ^3^ Department of Cardiology University of Wisconsin Madison WI USA; ^4^ Department of Population Health University of Wisconsin Madison WI USA; ^5^ Department of Biomedical Sciences Burrell College of Osteopathic Medicine Las Cruces NM USA; ^6^ Stead Family Department of Pediatrics University of Iowa Iowa City IA USA; ^7^ The John Rankin Laboratory of Pulmonary Medicine University of Wisconsin Madison WI USA; ^8^ Department of Kinesiology University of Wisconsin Madison WI USA; ^9^ Department of Biomedical Engineering University of Wisconsin Madison WI USA

**Keywords:** hypertension, hypoxia, preterm, pulse wave velocity, vascular function

## Abstract

**Objectives:**

Adults born prematurely have an increased risk of early heart failure. The impact of prematurity on left and right ventricular function has been well documented, but little is known about the impact on the systemic vasculature. The goals of this study were to measure aortic stiffness and the blood pressure response to physiological stressors; in particular, normoxic and hypoxic exercise.

**Methods:**

Preterm participants (*n* = 10) were recruited from the Newborn Lung Project Cohort and matched with term‐born, age‐matched subjects (*n* = 12). Aortic pulse wave velocity was derived from the brachial arterial waveform and the heart rate and blood pressure responses to incremental exercise in normoxia (21% O_2_) or hypoxia (12% O_2_) were evaluated.

**Results:**

Aortic pulse wave velocity was higher in the preterm groups. Additionally, heart rate, systolic blood pressure, and pulse pressure were higher throughout the normoxic exercise bout, consistent with higher conduit artery stiffness. Hypoxic exercise caused a decline in diastolic pressure in this group, but not in term‐born controls.

**Conclusions:**

In this first report of the blood pressure response to exercise in adults born prematurely, we found exercise‐induced hypertension relative to a term‐born control group that is associated with increased large artery stiffness. These experiments performed in hypoxia reveal abnormalities in vascular function in adult survivors of prematurity that may further deteriorate as this population ages.

## Graphical abstract

The long-term consequences of prematurity are not well understood. We found that adult survivors of prematurity have increased blood pressure during exercise and evidence of arterial stiffening.

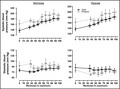

## 1 INTRODUCTION

Preterm birth confers a unique, long‐lasting physiological phenotype, including reduced pulmonary function (Lovering et al., 2013; Vrijlandt, Gerritsen, Boezen, Grevink, & Duiverman, 2006), impaired gas exchange during exercise (Farrell et al., 2015; Narang, Bush, & Rosenthal, 2009), and alveolar simplification, evidenced by reports of obstructive lung disease (Narang, 2010) and computed tomographic evidence of emphysema (Aukland et al., 2009; Wong et al., 2008). We previously reported that prematurely born adults have altered physiological responses to exercise and hypoxia that could not have been predicted from resting measures alone. Farrell et al. reported that the alveolar‐arterial oxygen difference is higher in preterm adults performing cycle exercise, despite similar maximal oxygen consumption and an augmented gas exchange efficiency during hypoxic exercise (Farrell et al., 2015). The fact that addition of hypoxia diminished the difference in gas exchange inefficiency and highlights the value of this added stress in probing mechanisms of physiological dysfunction. The hypoxic ventilatory drive is blunted in premature adults despite normal eupneic ventilation (Bates, Farrell, & Eldridge, 2014) and may be the result of impaired carotid body development (Bates, Welch, Randall, Petersen‐Jones, & Limberg, 2018). Adults born prematurely also experience delayed heart rate recovery after exercise (Haraldsdottir et al., 2019) and adolescents demonstrate altered heart rate variability (Haraldsdottir et al., 2018). Taken together, these findings suggest that exercise and hypoxia are both uniquely valuable in probing differences in the physiological response to stress, even in preterm individuals that appear physiologically indistinguishable from control, term‐born peers at rest.

These areas of research are important because very little is known as to how prematurely born adults will age. The survival of premature infants increased dramatically in the early 1990s with the introduction of surfactant, and the first cohort of surfactant‐era patients are only now approaching 30 years old. Still, very little is known about the long‐term impact of prematurity on systemic vascular function. Systolic and diastolic blood pressures are higher in premature children and this persists into adulthood (Edwards et al., 2014; Jong, Monuteaux, van Elburg, & Gillman, 1979; Luu, Katz, Leeson, & Thébaud, 2016). Elevated blood pressures may be the result of premature vascular stiffening that persists from birth. Tauzin et al. found that low birth weight infants had decreased aortic compliance compared to normal birth weight infant (Tauzin et al., 2006), although there are no data in the general premature population. Cheung et al. investigated 8‐year‐olds who were born prematurely and found birth weight and carotid‐femoral pulse wave velocity (PWV), reflecting aortic stiffness, are inversely correlated such that children born smaller had higher PWV (Cheung, Wong, Lam, & Tsoi, 2004). Increased PWV is associated with increased systolic blood pressure during exercise (Thanassoulis et al., 2012). Therefore, if increased arterial stiffness is the major contributor to increased blood pressure in this population, we would expect to observe increased blood pressure with exercise, with and without the addition of hypoxia.

The purpose of this study was to evaluate the dynamic blood pressure response to exercise and hypoxic stress in our well‐described population of preterm adults and evaluate arterial stiffness. We further hypothesized that PWV would be higher in preterm adults and, therefore, adults who were born prematurely would have an exaggerated blood pressure response to exercise compared to the term‐born adults. Consistent with our hypothesis, prematurely born adults had increased arterial stiffness and increased systolic and diastolic blood pressures during exercise. Contrary to our hypothesis, the addition of hypoxia to exercise caused a transient decrease in diastolic blood pressure in premature adults, while systolic blood pressure remained increased. This suggests that alterations in vascular tone and arterial stiffening both contribute, causing increased systolic and diastolic blood pressure during exercise in prematurely born individuals.

## 2 METHODS

### 2.1 Subject population and screening

All participants gave informed consent prior to participation. This protocol was approved by the Institutional Review Board at the University of Wisconsin and conducted in accordance with the Declaration of Helsinki**.** Preterm subjects (*n* = 10) were recruited from the Newborn Lung Project, a longitudinal cohort at the University of Wisconsin (Madison, WI), that registered premature patients admitted to nine neonatal intensive care units between 1989 (presurfactant era) and 1991 (surfactant era). This population has been described extensively (Evans, Palta, Sadek, Weinstein, & Peters, 1998; Farrell et al., 2015; Hagen, Palta, Albanese, & Sadek‐Badawi, 2006; Palta et al., 1990, 1996, 2001; Palta, Gabbert, Weinstein, & Peters, 1991; Palta, Sadek, Barnet, et al., 1998; Palta, Sadek, Lim, Evans, & McGuinness, 1998; Palta, Sadek‐Badawi, Evans, Weinstein, & McGuinnes, 2000; Palta, Sadek‐Badawi, Madden, & Green, 2007; Palta et al., 1994; Peppard et al., 2013; Weinstein, Peters, Sadek, & Palta, 1994; Young et al., 1997, 2009), but the hypothesis was developed a priori to data analysis. Patients recruited to this cohort were low birth weight (<1,500 g) and had a gestational age less than 36 weeks (mean, 28 ± 2 weeks). All preterm‐born subjects studied here received supplemental oxygen at birth and were mechanically ventilated. A term‐born, aged‐matched control population (*n* = 12) was recruited from the general public of Madison, WI.

Prior to participation, preterm‐ and term‐born individuals participated in a screening visit where they received a general medical screening exam, an echocardiogram overseen by a licensed echocardiographer (KRB) with measures of cardiac anatomy, pulmonary function tests (Farrell et al., 2015), and a graded, maximal exercise test on a cycle ergometer (Velotron, Quarq Technology), as has previously been described (Bates, Farrell, Drezdon, et al., 2014; Farrell et al., 2015). During the screening exercise test, participants breathed through a mouthpiece and wore nose clips, and the workload was increased in 50 W increments until the participant could no longer maintain a cadence >55 RPM despite vigorous verbal encouragement. The test was completed within 10 min (Buchfuhrer et al., 1983). Ventilation and metabolism were measured (AD Instruments, Exercise Physiology Package with Gemini Gas Analyzer) and VO_2_max was determined as the highest 20s value of oxygen consumption achieved (Poole, Wilkerson, & Jones, 2008). Control populations were well matched for body mass index (BMI), age, pulmonary function, and exercise capacity. Both populations had no prior history of cardiopulmonary disease, took no regular medication except hormonal birth control, and were nonsmokers.

### 2.2 Experimental design

Each participant completed two research visits. On one occasion, participants breathed 21% O_2_, balanced in N_2_, during the resting, baseline phase, and exercise protocol. On the other occasion, participants breathed 12% O_2_, balanced in N_2_ (Airgas, 12% ± 0.1% verified by mass spectroscopy). Participants were blinded as to the gas condition. Upon reporting to the laboratory, a 3F catheter was placed in the brachial arterial under local anesthesia and an intravenous catheter was placed in an antecubital vein. The arterial catheter was attached to a clinical grade pressure transducer (Edwards Lifesciences). A temperature sensing pill was swallowed for the measurement of core temperature (CoreTemp, HQInc).

Participants then sat on the same magnetically braked ergometer used for maximal exercise testing and donned nose clips and a mouthpiece for breath‐by‐breath measures of ventilation and metabolism. They remained seated quietly for at least 5 min until blood pressure and heart rate stabilized. Participants then began cycling at 50 W and the workload was increased 10 W every 2 min. Unlike the traditional exercise test used for screening, this prolonged graded exercise test was chosen in order to allow us to resolve subtle differences between the groups. The exercise protocol was terminated when participants could not maintain a cadence ≥55 RPM, despite strong verbal encouragement. Arterial blood gases were measured at rest and every other workload. The experiments described here were part of a larger project and the blood gas results have been previously reported (Farrell et al., 2015).

### 2.3 Heart rate, blood pressure, and pulse wave velocity

A multipoint calibration was performed for each transducer at the end of the study using a clinical transducer calibration device (Veri‐Cal, Utah Medical) that was verified monthly against a water manometer. The calibration of the transducer was performed at the end of the experiment in order to maintain the sterility of the arterial line.

Heart rate, derived from the arterial pressure waveform, and blood pressure recordings were collected for 5 min at rest and continuously throughout exercise (LabChart8 (AD Instruments). The final 45s of each stage was used for the analysis of blood pressure and heart rate. Aortic pulse wave velocity was derived from the intrabrachial artery pressure waveforms obtained during the resting phase as previously described (Pierce et al., 2013) (Hemolab, Harald Stauss Scientific). Briefly, brachial artery pressure waveforms were converted into central aortic pressure waveforms using a published transfer function (three‐parameter Windkessel model) as described by Sugimachi et al. (1997) and Sugimachi, Shishido, Miyatake, & Sunagawa (2001). The ascending aortic pressure waveforms were then decomposed into the forward and reflected wave components according to Westerhof, Guelen, Westerhof, Karemaker, & Avolio (2006) and Qasem & Avolio (2008) and the time delay between these two components was determined. Finally, the aortic pulse wave velocity was calculated by dividing the effective reflecting distance (EfRD), by the time delay between the forward and reflected waves. The EfRD was estimated using a previously validated equation: (EfRD = (0.173 × age) + (0.661 × BMI) + 34.548 cm) established by Pierce et al. (2013). This estimation of pulse wave velocity has been shown to agree with tonometry‐based measures.

### 2.4 Statistical analysis

Data are reported as mean ± *SE* unless otherwise indicated. Analyses were performed in Minitab (State College) and significance was set a priori at *p* < .05. Anthropometric, pulmonary function, and exercise capacity comparisons were made using a two‐sample *t* test. The impact of exercise and hypoxia on heart rate and blood pressure was evaluated using a repeated measure, nested multivariate linear model where the percent of maximal workload was the covariate, and the individual subject, group (preterm vs. term), baseline value of the parameter during nomoxic rest, and the gas (normoxia vs. hypoxia) were included as factors. An interaction term (group × gas) was also included in the analysis. Results of the multivariate linear model analysis are given in Table 4. Heart rate variability, PWV, and resting blood pressure values were evaluated with a two‐way analysis of variance for one independent (group) and one repeated (normoxia vs. hypoxia) measure model.

## 3 RESULTS

### 3.1 Anthropometric data, pulmonary function, and exercise capacity

The preterm and term‐born groups were well matched with no difference between gender, age, height, weight, body mass index (BMI), and body surface area (BSA) (Table 1). Mild preterm is classified as 32–36 weeks, very preterm as 28–31 weeks, and extremely preterm as < 28 weeks gestational age. The preterm groups had an average gestational age of 28 weeks, with 4 of the subjects being born at ≤28 weeks and the youngest being 25 weeks of gestation at birth (Moutquin, 2003). Average birth weight of the preterm group was 1,047 ± 95 g and one participant was small for gestational age at birth. Additional data from their neonatal intensive care unit stay are given in Table 1. The term‐born adults were all born at or after 36 weeks and weighed more than 1,500 g. Measures of cardiac anatomy, including right and left ventricular wall thickness, septal thickness, ejection fraction, end systolic and diastolic volumes, and left ventricular outflow tract diameter were within normal limits and were not different between groups.

**TABLE 1:** Anthropometric data of adults born prematurely and matched term‐born adults

	Term (*n* = 12)	Preterm (*n* = 10)	*p*‐value
Gender: M, F	4, 8	4, 6	
Age (y)	21 ± 0	21 ± 0	0.07
Height (cm)	170.7 ± 3.4	164.5 ± 3.9	0.25
Weight (kg)	71.7 ± 5.2	71.0 ± 6.8	0.94
BMI (kg/m^2^)	24.2 ± 1.1	25.4 ± 2.6	0.69
BSA (m^2^)	1.8 ± 0.1	1.8 ± 0.1	0.72
Gestational age (wk)	≥36	28 ± 1	
Birth weight (g)	≥1,500	1,047 ± 95	
Number of intubations		2 ± 1	
Ventilator time (h)		276 ± 68	
Length of stay in NICU (days)		74 ± 8	
Surfactant at birth: (Y,N)		6, 4	
Oxygen at 36 weeks: (Y,N)		5, 5	

Note

Data are presented as mean ± *SE*.

Abbreviations: BMI, body mass index; BSA, body surface area; *N*, no; NICU, neonatal intensive care unit; wk, weeks; Y, yes.

There was no significant difference between preterm and term‐born adults when comparing FEV_1_, FVC, FEV_1_/FVC, and DLCO (Table 2). Maximum wattage of the graded exercise test and VO_2_ max were also not different.

**TABLE 2:** Pulmonary function and exercise capacity

	Term (*n* = 12)	Preterm (*n* = 10)	*p*‐Value
FEV_1_ (L)	4.0 ± 0.3	3.5 ± 0.2	.19
FVC (L)	4.9 ± 0.4	4.5 ± 0.3	.38
FEV_1_/FVC	0.81 ± 0.03	0.77 ± 0.02	.28
DLCO (mL/min/mmHg)	27.5 ± 2.3	26.1 ± 1.7	.62
Maximum Wattage (W)	173 ± 14	145 ± 13	.18
VO_2_ Max (mL/min/kg)	35.8 ± 3.0	38.2 ± 1.9	.51

Note

Data are presented as mean ± *SE*. DLCO, diffusing capacity of the lung for carbon monoxide; FEV_1_, forced expiration volume in 1 s; FVC, forced vital capacity.

### 3.2 Heart rate and blood pressure

Aortic pulse wave velocity measured at rest was higher in the preterm group (*p* = .006) (Table 3) and was not altered by the addition of hypoxia (*p* = .059). Systolic and diastolic blood pressures were not different between groups in the period immediately before the onset of before exercise (Table 3). Normoxic, resting heart rate was higher in the preterm group (Table 3, *p* = .001).

**TABLE 3:** Pre‐exercise heart rate, blood pressure, pulse wave velocity, and heart rate variability parameters

	Term Born (*N* = 12)		Preterm (*N* = 10)	
	Normoxia	Hypoxia	Normoxia	Hypoxia
Heart rate (BPM)	87 ± 4	94 ± 4^†^	92 ± 3*	106 ± 3^†^
Systolic blood pressure (mmHg)	137 ± 7	145 ± 15	162 ± 14	152 ± 10
Diastolic blood pressure (mmHg)	80 ± 4	87 ± 10	92 ± 10	81 ± 7
Mean arterial pressure (mmHg)	103 ± 5	109 ± 11	118 ± 12	106 ± 8
Pulse pressure (mmHg)	57 ± 3	58 ± 7	70 ± 5*	71 ± 6
Aortic pulse wave velocity (m/s)	7.8 ± 0.3	8.8 ± 0.6	9.2 ± 0.8*	10.7 ± 1.0
Central augmentation index (%)	4.4 ± 2.2	2.9 ± 1.4	2.0 ± 1.2	0.5 ± 0.4
Average interbeat interval (ms)	709 ± 30	657 ± 24^†^	670 ± 26*	574 ± 20^†^
*SD* interbeat interval (ms)	57 ± 7	54 ± 9^†^	66 ± 11	35 ± 6^†^
*SD* rate (BPM)	7.1 ± 0.9	6.8 ± 0.9	8.1 ± 0.9	5.9 ± 0.6
VLF power (%)	36.1 ± 5.0	29.4 ± 4.5	34.4 ± 5.2	40.3 ± 5.9
LF power (%)	38.5 ± 4.4	38.3 ± 3.0	42.3 ± 4.8	42.8 ± 4.8
HF power (%)	25.2 ± 6.3	30.9 ± 4.1	23.8 ± 4	17.2 ± 4.0
LF/HF	3.5 ± 1.3	1.7 ± 0.4	2.4 ± 0.6	3.5 ± 0.7

Abbreviations: BPM, beats per minute; HF, high frequency; LF, low frequency; VLF, very low frequency.

* Represents an interaction of the group (*p* < .05).

^†^ Represents an effect of the gas (*p* < .05)

**FIGURE 1:**
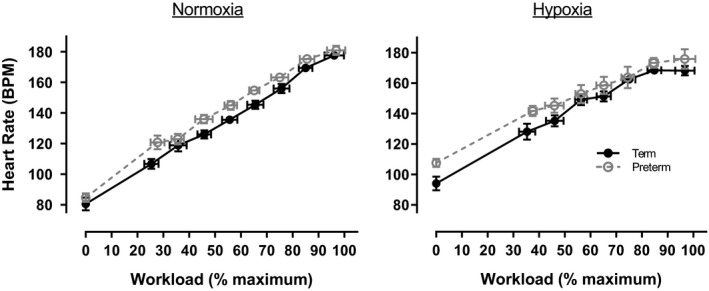
Heart rate during normoxic (21% oxygen) and hypoxic (12% oxygen) incremental exercise tests to volitional exhaustion in term‐born (*n* = 12) and preterm (*n* = 10) adults. Data are represented as mean ± *SE* and were analyzed with a repeated measures, nested multivariate model with the group (term vs. preterm), gas (normoxia vs. hypoxia), resting normoxic value, and an interaction (group × gas) included as factors. Statistical information is given in Table 4

In both groups, heart rate increased as a function of increasing workload (Figure 1, *p* < .001). Heart rate was consistently higher for the preterm group (group effect *p* < .001), even after considering significant differences in resting, normoxic heart rate (Table 4, *p* < .001). Hypoxia caused an increase in resting heart rate in both groups (Table 3, *p* < .001). We also observed a significant group × gas interaction effect (*p* = .01) that is likely the result of changes in resting and max heart rate. Hypoxia resulted in a 9 bpm reduction in maximum heart rate for the term‐born group, compared to a 6 bpm reduction in the preterm individuals. The term‐born group had a lower resting heart rate in hypoxia. Therefore, the overall increase in heart rate was 74 bpm compared to 68 bpm in the preterm group.

**TABLE 4:** Multivariate linear model of heart rate and blood pressure responses to normoxic and hypoxic exercise

Dependent variable	Independent variable	Regression coefficient (*β*)	Coefficient *SE*	*p*‐Value	Model adjusted *R* ^2^
Heart rate	Pre‐exercise normoxic value	.32	.05	**<.001**	83.8%
	Group	−2.84	.60	**<.001**	
	Gas	.38	.65	.56	
	Group × gas	1.55	.60	**.01**	
	Workload	.89	.02	**<.001**	
Systolic blood pressure	Pre‐exercise normoxic value	.97	.03	**<.001**	87.3%
	Group	−.13	.78	.87	
	Gas	−1.72	.69	**.013**	
	Group × gas	−.36	.71	.61	
	Workload	.40	.02	**<.001**	
Diastolic blood pressure	Pre‐exercise normoxic value	.81	.04	**<.001**	83.2%
	Group	−1.591	.50	**.002**	
	Gas	−3.55	.48	**<.001**	
	Group × gas	.91	.50	**.046**	
	Workload	.02	.02	.26	
Mean arterial pressure	Pre‐exercise normoxic value	.92	.04	**<.001**	86.1%
	Group	−1.84	.56	**.001**	
	Gas	−3.07	.52	**<.001**	
	Group × gas	.59	.54	.28	
	Workload	.15	.02	**<.001**	
Pulse pressure	Pre‐exercise normoxic value	1.31	.06	**<.001**	80.7%
	Group	2.42	.64	**<.001**	
	Gas	2.84	.52	**<.001**	
	Group × gas	10.5	.52	**.042**	
	Workload	.38	.02	**<.001**	

Bold indicates statistically significant values.

**FIGURE 2:**
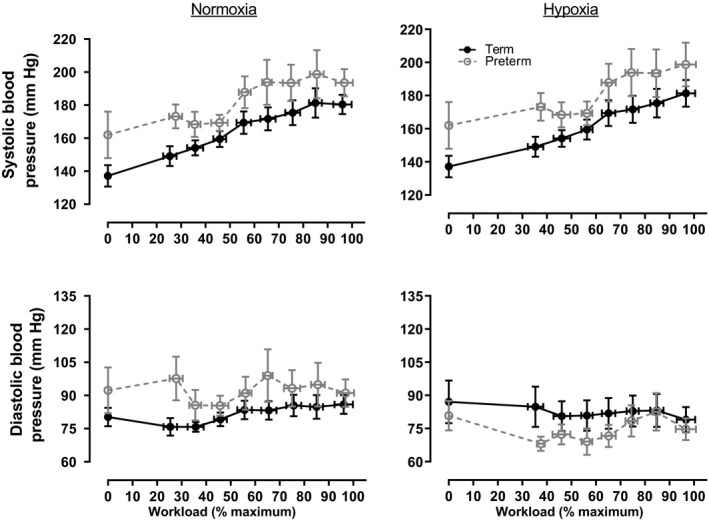
Systolic and diastolic blood pressure during normoxic (21% oxygen) and hypoxic (12% oxygen) incremental exercise tests to volitional exhaustion in term‐born (*n* = 12) and preterm (*n* = 10) adults. Data are represented as mean ± *SE* and were analyzed with a repeated measures, nested multivariate model with the group (term vs. preterm), gas (normoxia vs. hypoxia), resting normoxic value, and an interaction (group × gas) included as factors. Statistical information is given in Table 4

Systolic blood pressure increased as a function of increasing workload in both groups (Figure 2, *p* < .001) and was impacted by the addition of hypoxia (*p* = .013). Systolic blood pressure increased linearly in term‐born subjects performing normoxic exercise up until 90% of their maximal workload, while it plateaued at 60% of the maximal workload in preterm subjects. Both groups demonstrated a linear increase in systolic pressure with hypoxic exercise and, as in normoxia, systolic blood pressure was higher in the preterm group throughout the exercise protocol. Differences between preterm and term‐born individuals can be accounted for by pre‐exercise, systolic blood pressure (*p* < .001), suggesting that small differences in pre‐exercise blood pressure predict the higher systolic pressure during exercise.

**FIGURE 3:**
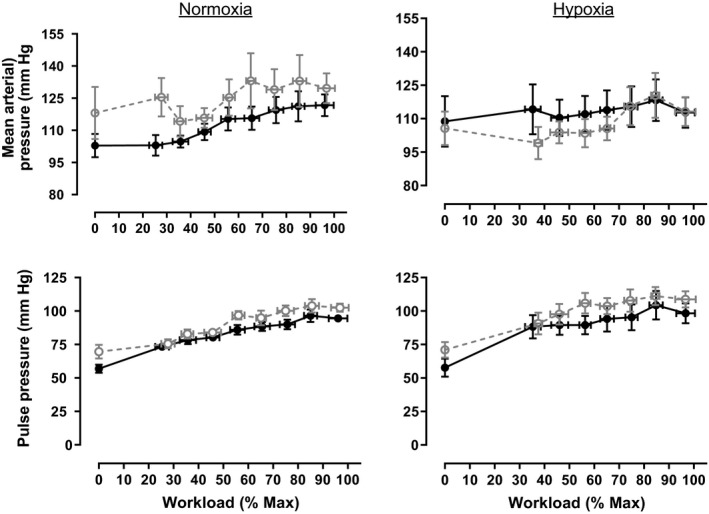
Mean arterial pressure and pulse pressure during normoxic (21% oxygen) and hypoxic (12% oxygen) incremental exercise tests to volitional exhaustion in term‐born (*n* = 12) and preterm (*n* = 10) adults. Data are represented as mean ± *SE* and were analyzed with a repeated measures, nested multivariate model with the group (term vs. preterm), gas (normoxia vs. hypoxia), resting normoxic value, and an interaction (group × gas) included as factors. Statistical information is given in Table 4. Mean arterial pressure was measured directly by averaging the brachial artery waveform

Consistent with classic studies of the effect of exercise on diastolic blood pressure (Hanson & Tabakin, 1965; Tabakin, Hanson, Merriam, & Caldwell, 1964), term‐born participants demonstrated little change in diastolic blood pressure during exercise in normoxia (Figure 2). Preterm individuals began the exercise at a higher diastolic pressure than the term‐born individuals and maintained a higher diastolic pressure until volitional maximum was approached (group effect, *p* = .002), even after considering effect of the normoxic, pre‐exercise diastolic pressure (*p* < .001). Diastolic pressure was increased by the addition of hypoxia in the term born. In contrast, exercise caused a reduction in diastolic pressure in the preterm population, and diastolic pressure was lower in the preterm group compared to controls throughout exercise (groups × gas effect *p* = .046). The differences in diastolic pressure largely contribute to the differences in mean arterial pressure (Figure 3). In normoxia, the mean arterial pressure was higher, consistent with increased systolic blood pressure (group effect *p* < .001). However, in hypoxia, the difference in mean arterial blood pressure between groups was reduced, driven by the reduction in diastolic pressure in the preterm group (gas effect, *p* < .001).

The pulse pressure is the difference between the systolic and diastolic blood pressure, depends on stroke volume and arterial stiffness (Lamia et al., 2007), and reflects the work of the left ventricle during ejection (Walley, 2016). In both groups, pulse pressure increased as a function of increasing workload (*p* < .001) and pulse pressure was higher in the preterm group compared to term‐born controls (*p* < .001). Hypoxia resulted in a higher pulse pressure in both groups (*p* < .001).

## 4 DISCUSSION

The major finding of this work is that healthy survivors of prematurity experience substantially higher blood pressures during exercise that are likely to have multiple mechanistic contributors. Whether prematurity directly impacts hypertension risk continues to be a topic of intense interest (Bertagnolli, Luu, Lewandowski, Leeson, & Nuyt, 2016). The overall purpose of this study was to evaluate the blood pressure response to exercise in an adult population of prematurely born adults. Crump, et al. described blood pressure in 636,000 adults, including 28,220 born prematurely, and found that young adults born prematurely (Lamia et al., 2007; Lewandowski Adam et al., 2015; Lovering et al., 2013; Luu et al., 2016; Martyn et al., 1995; Martyn & Greenwald, 1997; McEniery et al., 2011; Moutquin, 2003; Narang, 2010; Narang et al., 2009; Palta et al., 1990, 1991) had an increased risk of antihypertensive use (Crump, Winkleby, Sundquist, & Sundquist, 2011). Sipola‐Leppänen et al. (2015 measured 24‐hr ambulatory blood pressure and found that systolic blood pressure is ~5 mmHg higher in young adults born prematurely. While these studies suggest important baseline differences, the addition of a dynamic challenge allowed us to better interrogate vascular dysfunction in this population. This study adds to the field by evaluating the blood pressure response in the face of two physiologically relevant stressors – exercise and hypoxia.

### 4.1 What does the diastolic pressure in hypoxia tell us about the vascular function of preterm adults?

Increased blood pressure in premature individuals has been previously attributed to microvascular rarefaction (Bonamy, Martin, Jörneskog, & Norman, 2007) and increased circulating antiangiogenic factors (Lewandowski Adam et al., 2015). Notably, blood pressures were not correlated with capillary density. This begs the question whether increased vascular tone also contributes to abnormal blood pressure. Our data suggest that vascular reactivity is also impaired.

The use of exercise and hypoxia allows us to probe the hypothesis that vascular tone is also abnormal in the preterm population. In normoxic exercise, preterm adults demonstrate increased diastolic blood pressure, consistent with increased total peripheral vascular resistance and decreased microvascular density. However, the addition of hypoxia caused a decrease in blood pressure during exercise, suggesting that preterm‐born adults have an impaired vascular reactivity. Whether endothelial function is impaired in this population remains unknown. Bonamy, et al. evaluated endothelial function in the skin blood vessels of children born prematurely and found that the response to acetylcholine was not different than that of term‐born children (Bonamy et al., 2007). Furthermore, it is not known if endothelium‐independent vascular reactivity is altered. Yzydorczyk, et al. demonstrated that perinatal oxygen exposure impairs vascular reactivity to angiotensin II in an animal model of prematurity (Yzydorczyk et al., 2008). However, there are no studies as to the impact of prematurity on sympathetic tone or sympathetic vascular responsiveness and functional sympatholysis (Fadel, 2015).

### 4.2 What is the etiology of arterial stiffness in adult survivors of prematurity?

We derived aortic pulse wave velocity from brachial arterial waveforms obtained at rest with normoxic and hypoxic gas breathing. Our results indicate that aortic stiffness is elevated in adult survivors of prematurity. In general populations with matched blood pressures, age is the best predictor of aortic stiffness (Koivistoinen et al., 2007). Reusz, et al. measured aortic pulse wave velocity in 1,000 children and teenagers and noted that the 95th percentile for 20‐year‐old men and women is 7.5 and 7.0 m/s, respectively. Seven of ten of our preterm participants exceed the 95th percentile for their age group and six exceed the 95th percentile by > 1 m/s. Four individuals exceeded the 90th percentile for 70‐year‐old men and women (The Reference Values for Arterial Stiffness C, 2010), suggesting a severe premature vascular aging phenotype. We additionally observed an increased pulse pressure during exercise, which is likely related to the increased aortic stiffness. Given the value of pulse wave velocity as a biomarker for cardiovascular risk (Vlachopoulos, Aznaouridis, & Stefanadis, 2014), the elevated aortic PWV in preterm‐born adults potentially has important clinical implications, such as the need for a more rigorous assessment of cardiovascular risk in this population. We propose that increased pulse wave velocity may be a contributor to elevated systolic blood pressure during exercise, but note that we did not comprehensively evaluate arterial function and responsiveness.

The etiology of increased aortic stiffness in this preterm‐born adults is not well understood. One hypothesis for increased aortic pulse wave velocity is improper development of the aorta (Martyn et al., 1995). Elastin is deposited in the aorta primarily between 38 and 40 weeks of gestational age (Martyn & Greenwald, 1997). Preterm birth may disrupt this process, and it may be further inhibited by clinical treatment events. In animal models of prematurity, antenatal steroids (Bensley, De Matteo, Harding, & Black, 2011) and perinatal supplemental oxygen (Huyard et al., 2014) disrupt normal aortic development. It is noteworthy that all of our participants received supplemental oxygen at birth and we have described other physiological abnormalities that are likely driven by oxygen (Bates, Farrell, & Eldridge, 2014; Bates et al., 2018). While aortic pulse wave velocity has not been validated as a surrogate for aortic stiffness in hypoxia, we did not find it to be changed by hypoxia in either preterm‐ or term‐born individuals.

Alternatively, our observations may be described by premature aging. McEniery, et al. evaluated aortic pulse wave velocity in extremely prematurely born 11‐year‐olds and found that systolic and diastolic blood pressure were not different, and aortic pulse wave velocity was also not difference than that measured in term‐born peers (McEniery et al., 2011). Arterial wave reflections were elevated, suggesting the emergence of a phenotype. It is possible that an observable increase in aortic stiffness will only emerge as these children mature into young adulthood. None the less, the question of whether aortic stiffening occurs because of premature aging, disruption in vascular development, or a combination of both remains an unanswered question.

While we note increased aortic pulse wave velocity in the preterm population, we did not find an increase in the central augmentation index. The fact that the augmentation index does not change in the same direction as aortic pulse wave velocity in the preterm population is not necessarily surprising. While the augmentation index is influenced by arterial stiffness, it is not a pure measure of stiffness, per se. The augmentation index is impacted by additional factors including heart rate, vascular tone and geometry, height, and LV contractility (Hughes et al., 2013; Kelly, Millasseau, Ritter, & Chowienczyk, 2001; Sharman, Davies, Jenkins, & Marwick, 2009). Augmentation index is inversely proportional to heart rate (Wilkinson et al., 2000) and it is conceivable that increases in the augmentation caused by increased stiffness would be countermanded by the increased heart rate in the preterm population.

### 4.3 Why is heart rate elevated during exercise in survivors of prematurity?

Although peak oxygen uptake was not different between groups, heart rate was consistently higher during exercise in the premature group. Considering that the rate of oxygen uptake is similar between groups and is determined by both cardiac output and oxygen extraction (West, 2008), an increased heart rate suggests that either stroke volume or oxygen extraction is impaired. We found no statistically significant differences in left ventricular morphometry between groups, but cardiac function during exercise may be impacted by prematurity. Huckstep et al. evaluated left ventricle function during exercise and found that stroke volume was similar at baseline, but reduced at 60% and 80% of peak exercise. Cardiac output reserve was decreased in the preterm‐born group at each percent peak of exercise intensity (Huckstep et al., 2018). Consistent with our findings, prematurely born individuals may compensate for impairments in stroke volume with an increased heart rate (Goss et al., 2018). Additional comprehensive measures of hemodynamic function in this population are warranted.

### 4.4 Limitations

A strength of our study is invasive arterial measures of blood pressure during exercise, allowing us to obtain high fidelity pressure recordings and derive the aortic pulse wave velocity. Baseline arterial pressures were higher than might be expected in a young, healthy population. Despite the use of generous local anesthesia, we suspect the invasiveness of placing an arterial catheter may have influenced baseline blood pressure, resulting in higher baseline blood pressures than would be expected in a young adult population. Baseline blood pressure was also measured immediately before the onset of exercise, with the participant seated on the ergometer. No participant reported use of antihypertensive medication or a clinical diagnosis of hypertension and all participants had clinically normal blood pressure before the screening session (<130 mmHg systolic). We also used the brachial pressure tracings to derive the aortic pulse wave velocity based on a previously published algorithm (Pierce et al., 2013). Although this method has been validated against gold standard arterial tonometry, we appreciate that there is a small average disagreement between the two measures (0.04 ± 0.19 m/s), but no evidence of bias (Pierce et al., 2013). We are confident that the differences we observe here are not influenced by the absolute pulse wave velocity. Derivation of pulse wave velocity from the brachial arterial waveform has been validated in young, healthy individuals without cardiovascular disease. While prematurity was not included as a variable in these studies, it is reasonable to assume that this population likely included preterm individuals. Still, we note that the derivation of pulse wave velocity from a brachial waveform has not been specifically validated in survivors of prematurity. Our data provide a rationale for additional follow‐up studies in this area.

We did not directly measure cardiac output at rest or during exercise in this study. As we note, others have observed a diminished stroke volume and cardiac output reserve during exercise in a similar preterm population (Huckstep et al., 2018). This is physiologically consistent with our observation that the heart rate is higher in adult survivors of prematurity. To date, there have been no simultaneous measures of systemic hemodynamic function, inclusive of both cardiac output and blood pressure, in this population. Comprehensive studies of hemodynamic function, inclusive of cardiac output and blood pressure, are needed in this population.

Consistent with the populations of Wisconsin and Iowa, the participants in this study were white and nonhispanic. The results may not be broadly applicable. The study was derived from NICUs in Iowa and Wisconsin, which could elicit a geographical bias. Our study excluded any participants that were diagnosed with asthma, cardiovascular defects, and cognitive or motor defects, and all participants appeared clinically normal. We suspect that the invasiveness of the protocol and intensity of the exercise selected for high‐performing individuals and our data may not be applicable to patients with long‐term clinical sequelae. However, we observed altered cardiovascular responsiveness to exercise even in individuals who appear clinically normal. There may be individuals with a more severe phenotype that we did not characterize here.

### 4.5 Clinical Implications and conclusions

This is the first study to evaluate the dynamic blood pressure response to physiologically relevant stressors in adult survivors of prematurity. We found that preterm adults experience higher systolic blood pressures during exercise compared to a term‐born control group, and that this is possibly the result of increased large artery stiffness. The experiments performed in hypoxia revealed abnormalities in vascular function that may further deteriorate as this population ages. Preterm adults also require a higher heart rate during exercise, suggestive of alterations in stroke volume or oxygen extraction. Given the association between arterial stiffness and vascular dysfunction with cardiovascular diseases, our results suggest that the preterm population is vulnerable to cardiovascular diseases even in young adulthood. We propose that the addition of a cardiovascular stressor is critical to determine the full range of dysfunction in this population.

## CONFLICT OF INTEREST

The authors have no conflicts of interest to disclose.

## AUTHOR CONTRIBUTIONS

CRB, MP, ALS, ETF, KRB, MP, HMS, JMD, JS, GLP, MWE, and MLB designed the study; CRB, MP, ETF, KRB, MP, MWE, and MLB collected data; CRB, MP, ALS, HMS, JMD, JS, and MLB analyzed the data; CRB, MP, ALS, ETF, KRB, MP, HMS, JMD, JS, GLP, MWE, and MLB interpreted the results; CRB, MP, and MLB drafted the manuscript; and CRB, MP, ALS, ETF, KRB, MP, HMS, JMD, JS, GLP, MWE, and MLB edited and approved of the final manuscript.
